# The increase of osteopontin and β‐carboxy‐terminal cross‐linking telopeptide of type I collagen enhances the risk of hip fracture in the elderly

**DOI:** 10.1002/jcla.23204

**Published:** 2020-05-14

**Authors:** Jian‐Chun Lin, Zhong‐Guo Liu, Rui‐Ren Liu, Liang‐Wen Xie, Huang‐Lin Xie, He‐Guo Cai

**Affiliations:** ^1^ Department of Orthopaedics The Third Hospital of Xiamen Xiamen China

**Keywords:** bone mineral density, hip fracture in the elderly, osteopontin, β‐carboxy‐terminal cross‐linking telopeptide of type I collagen

## Abstract

**Background:**

Hip fracture in the elderly is a health burden worldwide due to its high mortality rate. This study was conducted to determine the possible mechanisms of osteopontin (OPN) and β‐carboxy‐terminal cross‐linking telopeptide of type I collagen (β‐CTX) in hip fracture in the elderly.

**Materials and Methods:**

In the study, we recruited 108 elderly patients with hip fracture diagnosed from May 2012 to May 2015 at the Third Hospital of Xiamen and 86 healthy individuals without a history of hip fracture were taken as controls. Serum levels of OPN and β‐CTX were then determined. The T and Z values for bone mineral density (BMD) were also measured. Moreover, logistic regression analysis was performed to assess the risk and protective factors for hip fracture in the elderly.

**Results:**

Serum levels of both OPN and β‐CTX were increased in elderly patients with hip fracture. OPN was positively correlated with β‐CTX. In addition, the levels of OPN and β‐CTX shared a positive association with the age, and a negative association with the BMD, in terms of T and Z values of the hip. In addition, increased BMD and outdoor sports might be protective factors for hip fracture, and an increase in levels of OPN and β‐CTX might be associated with a higher risk of hip fracture in the elderly population.

**Discussion:**

Collectively, increased serum levels of OPN and β‐CTX might be correlated with a higher risk of a hip fracture and have predictive values in the occurrence of hip fracture in the elderly.

## 1 INTRODUCTION

Hip fracture represents a serious health risks that frequently occurs in the elderly population.^1^ Hip fracture in the elderly always leads to severe functional impairment and deteriorating autonomy in daily life and is accompanied by high morbidity, mortality, functional disability and a decline in the quality of life.^2^, ^3^ Age has been shown to be a major risk factor, and the incidence of hip fracture is increasing with an aging population.^4^ Moreover, recent studies have reported that bone mineral density (BMD) and hip geometry along with the T‐ or Z‐score for BMD are also independently associated with an increased risk of hip fracture.^5^, ^6^ Although surgery has been recognized as the most effective treatment in all hip fractures, several outcomes, such as morbidity and mortality, are still not solved by this method.^7^ Thus, there is an unmet need to find an effective way to treat hip fracture in the elderly.

In hip fracture, osteopontin (OPN) is a protein made by bone tissues which plays a pivotal role in wound repair.^8^ OPN is a secreted protein expressed in bodily fluids and tissues and is considered an important factor in regulating bone mass and the toughness of bone.^9^ A recent study reported that OPN is overexpressed in idiopathic hip osteoarthritis and promotes its development.^10^ In addition, another study concluded that increased serum levels of OPN are correlated with prevalent fractures in postmenopausal women with type 2 diabetes.^11^ As a member of the carboxy‐terminal cross‐linking telopeptide of type I collagen (CTX) family, β‐CTX has been confirmed as a significant marker in bone resorption and formation.^12^ β‐CTX has been found to be significantly increased in patients with osteosarcoma, suggesting that it may have potential to act as a serum biomarker for the diagnosis of osteosarcoma.^13^ In addition, a study revealed that a high serum level of β‐CTX is an independent factor in aggravating hip fracture.^14^ Little is known about the synchronous role of OPN and β‐CTX in elderly patients with a hip fracture; herein, the study aims to identify potential mechanistic interplays between OPN and β‐CTX based on their serum levels in elderly patients with hip fracture.

## 2 MATERIALS AND METHODS

### 2.1 Ethics statement

All patients enrolled signed a written informed consent prior to sample collection. The study protocol was approved by the Ethics Committee of the Third Hospital of Xiamen. The study was conducted strictly adhered to the Declaration of Helsinki.

### 2.2 Study subjects

A total of 108 elderly patients diagnosed with hip fracture at the Third Hospital of Xiamen (23 men, 85 women; 60‐85 years old, with an average age of 69.02 ± 9.04 years) were enrolled in this study between May 2012 and May 2015. Meanwhile, 86 healthy individuals (24 men, 62 women; 58‐80 years old, with an average age of 67.06 ± 4.63 years) without a history of hip fracture were taken as controls. We collected the following information: age, gender, height, body mass, milk consumption, outdoor sports, smoking, and drinking habits and then calculated the body mass index (BMI) using the formula: BMI = weight (kg)/height (cm)^2^. Elderly patients were diagnosed with a hip fracture using X‐ray, computed tomography (CT), or magnetic resonance imaging (MRI) after histopathological examination or biopsy. The following patients were excluded: (a) patients with metabolic bone diseases; (b) patients with long‐term use of medicines, such as parathyroid hormone, that influence bone metabolism; (c) patients with recent traumatic fractures; (d) women prior to menopause or pregnant or lactating women; and (e) patients with serious heart, liver, kidney, or hematopoietic diseases.

### 2.3 BMD detection and osteoporosis (OP) diagnosis

The reduction of BMD is the major cause of fracture in the elderly. Spiral scanning was conducted using 64‐detector row spiral CT (Definition Flash, Siemens) and five solid phantom samples (Mindways Software). The image was transmitted to the Quanta Cloud Technology (QCT) workstation of Mindways Software in order to measure BMD. Scanning was carried out as follows: Patients were in a supine position, and the standard phantom was placed under the hip joints of patients. The midline of the phantom was overlapped with that of the patients. The scanning range was determined in the localization image. Spiral scanning was performed 5 cm from the upper acetabulum to the lower trochanter of the femur.

According to the diagnostic criteria issued by the World Health Organization (WHO), BMD of the first through fourth lumbar spine or femoral neck 1 standard deviation lower than the peak bone mass of healthy adults aged 20‐39 years was regarded as normal, 1‐2.5 standard deviations lower was regarded as osteopenia, and equal to or greater than 2.5 standard deviations was regarded OP. The measurements were expressed as a T‐score, and a T‐score ≥−0.1 was normal, −0.25 < T‐score <−0.1 was osteopenia, and T‐score ≤−2.5 was OP. In addition, a Z‐score >−2 represented a BMD in the normal range, and a Z‐score ≤−2 represented a BMD in the less than normal range.

### 2.4 Enzyme‐linked immunosorbent assay (ELISA)

In total, 8 mL fasting venous blood from patients in the early morning was collected, allowed to stand for 1.5‐2 hours, and then centrifuged at 2192 × *g* for 15 minutes. Next, approximately 5 mL serum was isolated and then placed in a disposable blood sampling cup at −70°C for detection. The samples of the fracture and control groups were tested after collection, and the serum samples were placed at room temperature for fusion before experiments. The levels of OPN and β‐CTX in the fracture and control groups were detected by ELISA, and the differences between the levels of OPN and β‐CTX in different genders were analyzed. The intra‐ and inter‐assay coefficients of variation (CVs) were <8% and <10%, respectively (ng/mL). The Elecsys 2010 automatic electrochemiluminescence immunoassay machine (Roche) and correlative kit were employed. The CVs were 4.3% and 5.8%, respectively (pg/mL), and the lower limit and upper limit were 10 pg/mL and 6000 pg/mL, respectively. Afterward, the samples were incubated, washed, and stained by 3,3′,5,5′‐tetramethylbenzidine (TMD) according to the kit instructions, and the absorbance (A) was measured at a wavelength of 450 nm using a microplate reader.

### 2.5 Statistical analysis

Statistical analysis was conducted using SPSS 21.0 software (IBM Corp). The counting data were expressed in percentages and analyzed by chi‐square test, while the measurement data were expressed as mean ± standard deviation. The normal distribution and homogeneity of variances were examined. Following this, two groups were compared using unpaired *t* test. If data failed to meet the criteria of normal distribution and homogeneity of variances, rank sum tests were conducted. Correlation among clinical parameters was analyzed by Pearson's correlation analysis. The independent diagnostic value of OPN and β‐CTX in predicting hip fracture in the elderly was evaluated using receiver operating characteristic (ROC) curves. Logistic regression analysis was used for independent risk factors for hip fracture. *P* < .05 was considered statistically significant.

## 3 RESULTS

### 3.1 BMD, T value, and Z value are decreased in elderly patients with hip fracture

The basic features of the fracture and control groups are displayed in Table 1. No significant differences were observed in gender, height, age, and tobacco and alcohol habits between the two groups (*P* > .05). The results of dual‐energy X‐ray absorptiometry (DEXA) showed the BMD as well as T value and Z value of the hip was significantly decreased in the fracture group compared to the control group (*P* < .05). Furthermore, we noted significant differences among milk drinking, outdoor sports, and history of fracture between the two groups (*P* < .05).

**Table 1:** Clinical parameters in the control group and the hip fracture group

Clinical parameters		Fracture group	Control group	*X^2^*	*P* value
Gender	Male	23 (21.30)	24 (27.91)	1.14	.286
	Female	85 (78.70)	62 (72.09)		
Height (cm)	<165 cm	54 (50.00)	45 (52.33)	0.104	.748
	≥165 cm	54 (50.00)	41 (47.67)		
Age (years)	<65	51 (47.22)	29 (33.72)	3.601	.058
	≥65	57 (52.78)	57 (66.28)		
BMD (T value)	T ≥ −1.0	30 (27.78)	9 (10.47)	9.092	.011
	−2.5 < T < −1.0	27 (25.00)	29 (33.72)		
	T ≤ −2.5	51 (47.22)	48 (55.81)		
BMD (Z value)	T > −2	41 (37.96)	51 (59.30)	8.744	.003
	T ≤ −2	67 (62.04)	35 (40.70)		
BMI	≤28.0 kg/m^2^	36 (33.33)	73 (84.88)	51.680	<.001
	>28.0 kg/m^2^	72 (66.67)	13 (15.12)		
Milk drinking	Yes	23 (21.30)	58 (67.44)	41.920	<.001
Sports	Yes	32 (29.63)	58 (67.44)	27.520	<.001
Smoking	Yes	56 (51.85	39 (45.35)	0.810	.368
History of fracture	Yes	77 (71.30)	32 (37.21)	22.600	<.001
A recent history of falling within 3 y	Yes	87 (80.56)	37 (43.02)	29.240	<.001
BMD (g/cm^2^)	‐	0.52 ± 0.24	0.67 ± 0.21	4.568	<.001

Note

The fracture group, n = 108; the control group, n = 86; count data were expressed as cases or percentage, and measurement data were expressed as mean ± standard deviation, analyzed by chi‐square test or independent‐sample *t* test.

Abbreviations: BMD, bone mineral density; BMI, body mass index.

### 3.2 Overexpressed serum levels of OPN and β‐CTX are found in elderly patients with hip fracture

**Figure 1:**
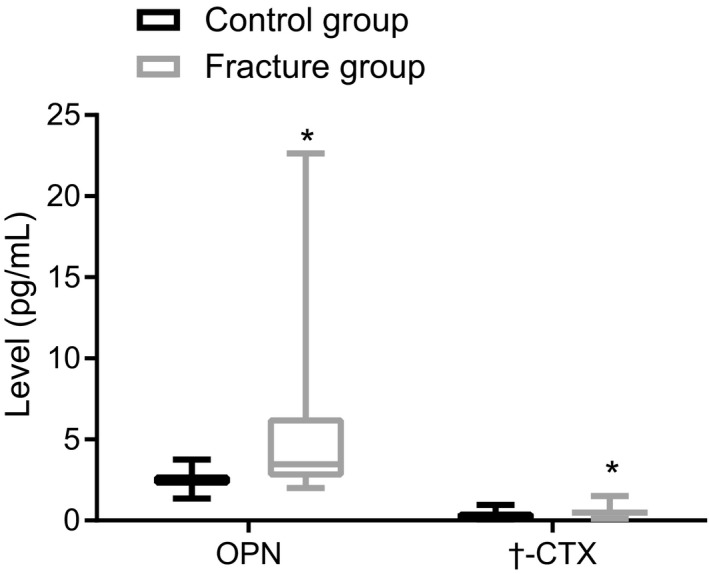
Osteopontin and β‐CTX are upregulated in elderly patients with a hip fracture. The fracture group, n = 108; the control group, n = 86. Measurement data were expressed as the mean ± standard deviation, analyzed by independent‐sample *t* test. *P < .05 vs the control group. OPN, osteopontin; β‐CTX, β‐carboxy‐terminal cross‐linking telopeptide of type I collagen

Next, we determined the serum levels of OPN and β‐CTX in elderly patients with hip fracture using an ELISA (Figure 1). Our results show that the serum levels of OPN (5.25 ± 3.59 vs 0.51 ± 0.28) and β‐CTX (2.46 ± 0.51 vs 0.28 ± 0.15) were significantly increased in the fracture group compared with the control group, respectively (*P* < .05). Taken together, elderly patients with hip fracture had elevated serum levels of OPN and β‐CTX.

### 3.3 OPN and β‐CTX positively correlate with age, while OPN and β‐CTX negatively correlate to BMD, T value and Z value in elderly patients with hip fracture

**Figure 2:**
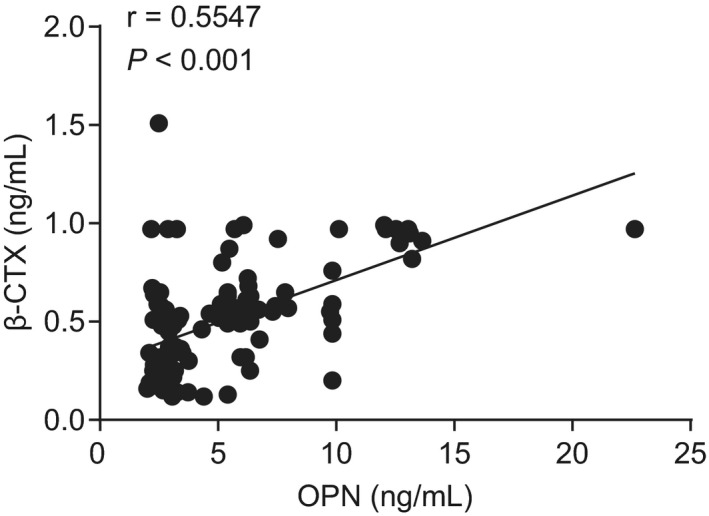
Serum levels of OPN and β‐CTX are in a positive correlation with the age of elderly patients with hip fracture. OPN, osteopontin; β‐CTX, β‐carboxy‐terminal cross‐linking telopeptide of type I collagen

A relative analysis was conducted to better understand the relationship of OPN and β‐CTX serum levels in relation to other hip fracture‐related risk factors. The results suggested a positive correlation between OPN and β‐CTX levels and age (*r* = .444, *P* < .001; *r* = .274, *P* = .004), and a negative correlation between OPN and β‐CTX levels and BMD (*r* = −.375, *P* < .001; *r* = −.198, *P* = .040), T value (*r* = −.209, *P* = .030; *r* = −.241, *P* = .012) and Z value (*r* = −.216, *P* = .025; *r* = −.224, *P* = .020) in the fracture group (Table 2). In addition, OPN was positively correlated with β‐CTX (*r* = .555, *P* < .001, Figure 2) in the fracture group. Herein, OPN and β‐CTX may increase the risk of hip fracture in the elderly.

**Table 2:** Serum levels of OPN and β‐CTX share a positive association with the age in old patients with hip fracture

	OPN		β‐CTX	
	*r*	*P*	r	*P*
Age	.444	＜.001	.274	.004
BMD	−.375	＜.001	−.198	.04
T value of hip	−.209	.03	−.241	.012
Z value of hip	−.216	.025	−.224	.02

Note

Correlation was analyzed by Pearson correlation analysis.

Abbreviations: BMD, bone mineral density; OPN, osteopontin; β‐CTX, β‐carboxy‐terminal cross‐linking telopeptide of type I collagen.

### 3.4 The occurrence of hip fracture in the elderly is associated with Z value, milk drinking, BMI, outdoor sports, history of fracture, recent history of falling within 3 years, BMD, and serum levels of OPN and β‐CTX

Next, we performed single‐factor logistic regression analysis for the clinical parameters in Table 1, including T value, Z value, BMI, milk drinking, outdoor sports, smoking, history of fracture, recent history of falling within 3 years, BMD, and serum levels of OPN and N‐CTX in elderly patients with a hip fracture. Our results shown in Table 3 revealed that the occurrence of hip fracture in the elderly was significantly correlated with the Z value, milk drinking, BMI, outdoor sports, history of fracture, a falling history in recent 3 years, BMD, and serum levels of OPN and β‐CTX. There was no significant correlation between the T value and smoking in the hip fracture group (Table 3).

**Table 3:** Single‐factor logistic regression analysis of risk factors for hip fracture in the elderly

Indicators	B	S E	Wald	*P*	OR (95% CI)
T value	0.118	0.078	2.302	.129	1.125 (0.966‐1.311)
Z value	−0.287	0.102	7.962	.005	0.750 (0.615‐0.916)
BMI	0.102	0.034	9.089	.003	1.107 (1.036‐1.183)
Milk drinking	−2.035	0.329	38.289	<.001	0.131 (0.069‐0.249)
Outdoor sports	−1.593	0.312	26.071	<.001	0.203 (0.110‐0.375)
Smoking	0.261	0.290	0.809	.368	1.298 (0.735‐2.290)
History of fracture	1.433	0.308	21.615	<.001	4.192 (2.291‐7.669)
A recent history of falling within 3 y	1.702	0.326	27.197	<.001	5.486 (2.894‐10.403)
BMD	−3.077	0.706	18.993	<.001	0.046 (0.012‐0.184)
OPN	1.523	0.319	22.787	<.001	4.587 (2.454‐8.574)
β‐CTX	5.314	0.984	29.152	<.001	203.082 (29.511‐1397.536)

Abbreviations: BMD, bone mineral density; BMI, body mass index; OPN, osteopontin; β‐CTX, β‐carboxy‐terminal cross‐linking telopeptide of type I collagen.

### 3.5 The increase in serum levels of OPN and β‐CTX is correlated with hip fracture in the elderly

Multiple stepwise logistic regression analysis was carried out to further characterize the indicators where significant differences were observed (Table 3). As shown in Table 4, increased serum levels of OPN and β‐CTX and a history of falling within 3 years were risk factors for hip fracture in the elderly (OR = 6.263 [2.608‐15.037], 31.296 [3.138‐312.125], 7.582 [2.702‐21.280], respectively). In addition, the increases of BMD and outdoor sports (OR = 0.050 [0.006‐0.510], 0.118 [0.044‐0.312]) appeared to be protective factors for hip fracture in the elderly. Accordingly, serum levels of OPN and β‐CTX may be related to the risk of hip fracture in the elderly.

**Table 4:** Multiple stepwise logistic regression analysis of factors for hip fracture in the elderly

Indexes	*B*	*S E*	*Wald*	*P*	*OR (95%CI)*
OPN	1.835	0.447	16.852	< 0.001	6.263 (2.608‐15.037)
β‐CTX	3.443	1.173	8.611	0.003	31.296 (3.138‐312.125)
BMD	‐2.861	1.116	6.568	0.010	0.050 (0.006‐0.510)
Outdoor sports	‐2.139	0.497	18.524	< 0.001	0.118 (0.044‐0.312)
Falling history in recent 3 y	2.026	0.527	14.803	< 0.001	7.582 (2.702‐21.280)
Constants	‐4.953	1.432	11.965	0.001	0.007

Note

Data were analyzed using logistic regression analysis.

Abbreviations: BMD, bone mineral density; CI, confidence interval; OPN, osteopontin; OR, odds ratio; β‐CTX, β‐carboxy‐terminal cross‐linking telopeptide of type I collagen.

### 3.6 OPN and β‐CTX are sensitive predictors of hip fracture in the elderly

**Figure 3:**
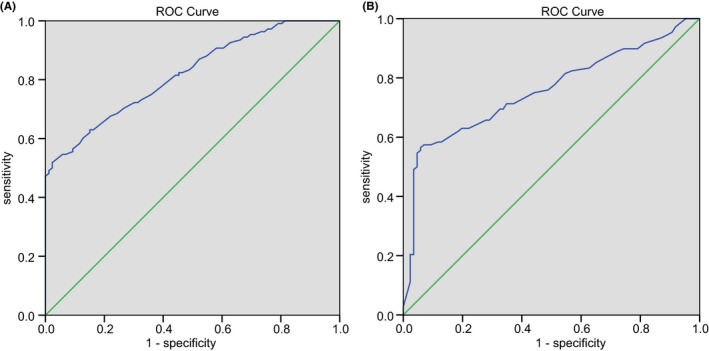
Osteopontin and β‐CTX are predictors for the risk of a hip fracture in the elderly. ROC, receiver operating characteristic; OPN, osteopontin; β‐CTX, β‐carboxy‐terminal cross‐linking telopeptide of type I collagen

Receiver operating characteristic curves were used to evaluate the independent diagnostic value of OPN and β‐CTX in hip fracture in the elderly. The area under the curve of patients with OPN and β‐CTX was 0.818 and 0.757, respectively (*P* < .05; Table 5, Figure 3). Taken together, we considered that OPN and β‐CTX might be reference factors in predicting the risk of hip fracture in the elderly.

**Table 5:** Osteopontin and β‐CTX are crucial indicators in evaluating the risk of hip fracture in old age

ROC curve	Area under curve	95% CI	*P*
β‐CTX (HF)	0.818	0.761‐0.875	<.001
OPN (HF)	0.757	0.688‐0.825	<.001

Note

Data were analyzed using ROC.

Abbreviations: CI, confidence interval; HF, hip fracture; OPN, osteopontin; ROC, receiver operating characteristic curve; β‐CTX, β‐carboxy‐terminal cross‐linking telopeptide of type I collagen.

## 4 DISCUSSION

Hip fracture is considered to be a major cause of high morbidity and mortality among elderly people.^15^ According to a recent study, serum albumin can be used to predict the clinical prognosis of hip fracture in the elderly.^16^ Our study determined the predictive potential of serum levels of OPN and β‐CTX in hip fracture in the elderly. Collectively, experimental results suggest that serum levels of OPN and β‐CTX are independent predictors for the risk of hip fracture. Serum levels of OPN and β‐CTX were positively correlated with hip fracture, and increased serum levels of OPN and β‐CTX could increase the risk of hip fracture in the elderly.

Our ELISA results showed significantly higher serum levels of OPN and β‐CTX in hip fracture in the elderly compared to healthy controls. Previous studies have shown that a high serum level of OPN results in increased fracture and a worse lipid profile in postmenopausal women with type 2 diabetes.^11^ Higher serum levels of OPN are observed in patients with OP and are correlated with the decline of BMD, increase of bone turnover markers, and osteoporotic vertebral fractures.^17^ Furthermore, OPN downregulation plays a role in fracture repair by regulating vascularization, matrix organization, and mechanical strength while facilitating late stage bone remodeling.^18^ Moreover, serum levels of OPN are observed to be significantly increased in the elderly population (over 75 years old) with hip fracture, suggesting that OPN is an important predictor for the risk of hip fracture in the elderly.^19^ OPN is expressed in bone cells, such as osteoblasts and osteocytes, and it shares an association with bone turnover and BMD in postmenopausal women.^20^

Carboxy‐terminal cross‐linking telopeptide of type I collagen is a significant marker of bone metabolism.^21^ Urinary CTX‐II is closely correlated with the dynamic bone turnover of knees signified by scintigraphy, thereby contributing to joint space narrowing and osteophyte severity along with progression of osteophytes in osteoarthritis.^22^ In addition, a recent study pointed out that increased serum levels of β‐CTX can be considered a potential marker for predicting the risk of hip fracture, since β‐CTX is negatively correlated with total hip BMD.^23^ Moreover, β‐CTX is a significant factor in predicting bone metastasis in lung cancer, as the serum levels of β‐CTX are significantly higher in patients with more than 3 bone metastases compared to patients with <3 bone metastases.^12^ The results from our multivariate analysis suggest that increased serum levels of OPN and β‐CTX also correlate with an enhanced risk of hip fracture in the elderly. The abovementioned observations indicate that OPN and β‐CTX may be used as potential markers in predicting the risk of hip fracture in the elderly.

Another important finding was that the serum levels of OPN and β‐CTX positively correlated with age, while they shared a negative correlation with BMD, the T value and, the Z value of the hip. One study identified increasing age as a major factor contributing to the enhancement of hip fracture risk, as increased age can also decrease the BMD index.^24^ Furthermore, another study showed that age has a positive correlation with the serum level of OPN, whereby the levels of OPN increase with age which leads to attenuated skeletal muscle regeneration in elderly people.^25^ Moreover, Pan F H et al concluded that the serum level of β‐CTX increases with age in women, as the serum level of β‐CTX was significantly higher in the groups that contained patients older than 50 years old compared to the younger than 49‐year‐old groups.^26^ BMD is considered to be a significant biomarker for accumulative exposure to multiple factors, such as estrogen, vitamin D, calcium, and physical activity.^27^ A study shows that loss of BMD results in the morbidity of hip fracture.^28^ Moreover, OPN downregulates BMD, while it upregulates the level of bone turnover markers along with osteoporotic vertebral fractures in postmenopausal women.^17^ Evidence suggests that BMD negatively correlates to OPN and β‐CTX at the lumbar spine (*r* = −.38, *P* = .002 and *r* = −.30, *P* = .02, respectively).^29^ Therefore, we propose that elevated levels of OPN and β‐CTX may increase the risk of hip fracture in the elderly through their constant action on reducing BMD.

In summary, the findings from this study demonstrate that serum levels of OPN and β‐CTX are significant risk factors for hip fracture, and higher levels of OPN and β‐CTX are associated with an enhanced risk of hip fracture in the elderly. Therefore, OPN and β‐CTX can potentially function as risk factors for the prognosis of hip fracture in the elderly as well as a target for the prevention of hip fracture in the elderly. Nevertheless, more experiments are necessary to provide results that can further elucidate the role of OPN and β‐CTX in the risk of hip fracture in the elderly. In addition, a large sample size, longer observation times, and funding are needed in prospective longitudinal cohort studies. Unfortunately, our cohort for this study was too limited in scope to complete a prospective longitudinal study. Therefore, a study with a larger sample size will be needed to conduct a prospective longitudinal cohort study to ensure the accuracy of the present study.

## CONFLICT OF INTEREST

The authors declare that they have no competing interests.

## AUTHORS' CONTRIBUTIONS

Zhong‐Guo Liu and Jian‐Chun Lin designed the study. Liang‐Wen Xie collated the data, carried out data analyses, and produced the initial draft of the manuscript. Jian‐Chun Lin, Huang‐Lin Xie, He‐Guo Cai, and Rui‐Ren Liu contributed to drafting the manuscript. All authors have read and approved the final submitted manuscript.

## ETHICAL APPROVAL

All patients enrolled signed written informed consents prior to sample collection. The study protocol was approved by the Ethics Committee of the Third Hospital of Xiamen. The study was conducted strictly adhered to the Declaration of Helsinki.
